# Supplementary material for: MetaRibo-Seq measures translation in microbiomes
Source: Nat Commun. 2020 Jun 29;11:3268. doi: 10.1038/s41467-020-17081-z (PMC7324362; doi:10.1038/s41467-020-17081-z)
Supplement: Supplementary file 10 — Supplementary Data 7 [file 41467_2020_17081_MOESM10_ESM.zip › File2/Confidence_High_Taxonomy/421981_out.krona.html]

Javascript must be enabled to view this page.

members
magnitude
magnitudeUnassigned
count
unassigned
taxon
rank

421981\_out

35

35
2
superkingdom

1
976
phylum

1
200643
class

171549
1
order

1
815
family

1
816
genus


SRS147139\_contig\_number\_31432
817
1
species

1239
34
phylum

34
186801
class

order
34
186802

family
541000
1

genus
1
216851

species
1
853

SRS050941\_contig\_number\_13114

33
186803
family

genus
841
1

species
1

SRS148159\_contig\_number\_17600
166486

1407607
12
genus


SRS051882\_contig\_number\_17526SRS064276\_contig\_number\_21274SRS1041036\_contig\_number\_contig-100\_226.227SRS104311\_contig\_number\_43158SRS1055043\_contig\_number\_22076SRS1055049\_contig\_number\_64SRS144183\_contig\_number\_9332SRS144714\_contig\_number\_25247SRS147346\_contig\_number\_33653SRS147919\_contig\_number\_16099SRS148721\_contig\_number\_26273SRS893378\_contig\_number\_8753
1150298
12
species

4
genus
20
572511

SRS015431\_contig\_number\_78745SRS017103\_contig\_number\_2812SRS075821\_contig\_number\_19137SRS1041136\_contig\_number\_8746

species
6

SRS019068\_contig\_number\_15717SRS062654\_contig\_number\_23484SRS098644\_contig\_number\_35389SRS1041145\_contig\_number\_33710SRS104636\_contig\_number\_17193SRS144135\_contig\_number\_30770
2292986

species
2292959

SRS077454\_contig\_number\_18181SRS077502\_contig\_number\_contig-100\_308.113172
2

2292962

SRS050422\_contig\_number\_21498SRS097889\_contig\_number\_34649
2
species

species

SRS097920\_contig\_number\_contig-100\_272.142996
2292990
1

1

SRS077392\_contig\_number\_21276
2292960
species

species
3
1896976

SRS022071\_contig\_number\_21714SRS063127\_contig\_number\_2387SRS142542\_contig\_number\_14368

1

SRS049773\_contig\_number\_17619
1262757
species
